# The Role of Physical Exercise in Sexual Health and Body Image in Women Living with and Surviving Breast Cancer: A Scoping Review

**DOI:** 10.3390/healthcare13070741

**Published:** 2025-03-26

**Authors:** Carmen Giulia Lia, Francesca Greco, Alessandra D’Amato, Maria Grazia Tarsitano, Gian Pietro Emerenziani, Federico Quinzi

**Affiliations:** 1 Department of Clinical and Experimental Medicine, Magna Græcia University of Catanzaro, 88100 Catanzaro, Italy; carmengiulia.lia@studenti.unicz.it (C.G.L.); f.greco@unicz.it (F.G.); alessandra.damato@studenti.unicz.it (A.D.); emerenziani@unicz.it (G.P.E.); fquinzi@unicz.it (F.Q.); 2 Department of Human Science and Promotion of Quality of Life, San Raffaele Open University of Rome, 00166 Rome, Italy

**Keywords:** sexual function, physical activity, quality of life, breast neoplasm

## Abstract

**Background/Objective.** Breast Cancer (BC) is a growing medical concern that may heavily influence sexual functioning (SF) and body image (BI) in BC patients. In healthy individuals, physical exercise (PE) has been proposed as a crucial factor to improve BI. However, little is known about the effects of PE on BI and SF in BC patients. Therefore, the aim of this study is to summarize the extant literature regarding the effects of PE on BI and SF in these populations. **Methods.** Our review, conducted using the PRISMA extension for a Scoping Review, was carried out in three databases: PubMed, Scopus and Web of Science. Only Randomized Control Trials (RCT) evaluating the effects of different types of PE on BI and/or SF in a population affected by or who have survived BC were included. **Results.** The literature search yielded 488 studies. Twelve studies resulted in being eligible for inclusion in this review. The effects of aerobic exercise on BI and SF were scant. Studies employing resistance training as PE provided contrasting results. Conversely, studies using holistic approaches yielded larger benefits on BI and SF. **Conclusions.** Our results showed that PE has marginal effects on BI and SF. PE interventions longer than six months and based on holistic activities should be implemented to improve BI and SF in BC patients. To safely draw conclusions on the effects of PE on BI and SF, future studies should consider more accurate monitoring of exercise intensity, and a thorough evaluation of the possible mediators of the effects of PE in these populations.

## 1. Introduction

Breast cancer (BC) is a pathology caused by an uncontrolled growth of breast cells [1]. BC more often and more significantly affects women’s health [2]. A recent study on the epidemiology of cancer worldwide showed that breast cancer accounted for approximately 24% of the 9.7 million new cases of cancer in 2022, resulting in approximately 2.3 new cases per year [2]. Moreover, the same study showed that breast cancer accounts for 15.4% of cancer-related deaths [2]. In the last forty years in highly developed countries, age-standardized BC mortality registered a decrease of 40% [1]. This drop in BC-related mortality in females may be attributed to the evolution of screening technologies and to the greater accuracy in the diagnostic phase [3]. The reduction in BC-related deaths calls the scientific and clinical world to face new issues related to the larger survival rate of BC patients. Among these, the enhancement of Quality of Life (QoL) in females surviving BC is of paramount importance and in recent decades has become one of the prioritized goals of BC treatment [4]. Health-related QoL can be defined as BC patients’ perception of how their diagnosis, treatment, post-treatment, and survivorship may influence their physical, mental and social health [4]. The above-mentioned definition encompasses the three dimensions of the health concept as stated by the WHO: social, mental and physical domains. Several indicators contribute to the assessment of each of the above-mentioned dimensions. Sexual functioning (SF) is a fundamental indicator as it influences all aspects of health [5]. According to Rosen et al. [6], SF is composed of five domains: desire and subjective arousal, lubrication, orgasm, satisfaction, and pain or discomfort. The proper functioning of sexuality is necessary for a positive perception of QoL [7]. A particular construct of the psychological field which affects SF seems to be body image (BI) [8]. Body image can be defined as the self-perception of one own’s body, which should be interpreted from an emotional and cognitive standpoint [9,10]. Women, in general, may be particularly exposed to BI-related issues [8]. BC, due to the modifications it causes in physical, psychological and social fields, has the potential to modify BI, self-perception and may lead to the avoidance of sexual activity [7,11,12].

Different types of interventions have been proposed to improve the perception of BI and consequently enhance the satisfaction related to SF in BC patients. Some interventions do not focus their actions specifically on the reproductive system but rather may influence psychological aspects (cognitive behavioural therapy [13]; or peer counselling programmes [14]). These approaches could contrast with sexual dysfunction-related stress and improve women’s connection with their sexuality. Otherwise, interventions which have local applications are suggested to improve SF. Specifically, laser treatments [15], vaginal hormonal therapy [16], moisturizers [17], oil application [18] and pelvic floor exercise [19] are proposed to diminish the dyspareunia that is consequent to BC treatments. Among these interventions, physical activity (PA), defined as any bodily movement produced by skeletal muscles that results in energy expenditure [20], seems particularly beneficial to counteract the negative effects of cancer and its treatments on functional levels, anxiety, depression and social functioning [21]. Specifically, physical exercise (PE), which can be defined as a structured, planned and repetitive type of PA [20] aiming at improving or maintaining one or more components of physical fitness, has been reported to improve QoL, decreasing depression, stress and anxiety and enhancing emotional feelings in oncologic patients [21]. Among the multiple forms of PE, aerobic exercise seems to be the optimal exercise modality to improve QoL [22] as it may reduce cancer-related fatigue [21], whereas holistic disciplines (i.e., yoga) seem effective to improve QoL and reduce depression [23].

It has been demonstrated that PE may influence several components of physical fitness in BC patients [24]. Muscular fitness and muscular strength may benefit from resistance exercise or Pilates. Further, bone mineral density, flexibility and body composition, in terms of fat mass reduction, can be positively influenced by these two intervention modalities [22,24,25].

Considering the positive effects of PE on multiple BC-related issues, the present review aims at investigating the effects of PE on two specific sub-domains of QoL, SF and BI, in women affected by and who have survived BC.

## 2. Methods

Our study was conducted using the Preferred Reporting Items for Systematic review and Meta-Analysis (PRISMA) extension for scoping reviews (PRISMA-ScR) [26].

At the beginning of October 2024, two authors defined the search strategy and the main topic of this scoping review: breast neoplasm, exercise, body image and sexual health were chosen as keywords. A literature search was carried out by one independent reviewer between October and November 2024 (last accessed 24 January 2025) in three databases: PubMed, Scopus and Web of Science (Medline). The results of the research strategies were exported as research information system (RIS) files for the Web of Science and Scopus databases, and as comma-separated value (CSV) files for the PubMed database. Table 1 shows the research queries used and the number of studies identified from the literature search for each database.

**Figure 1:**
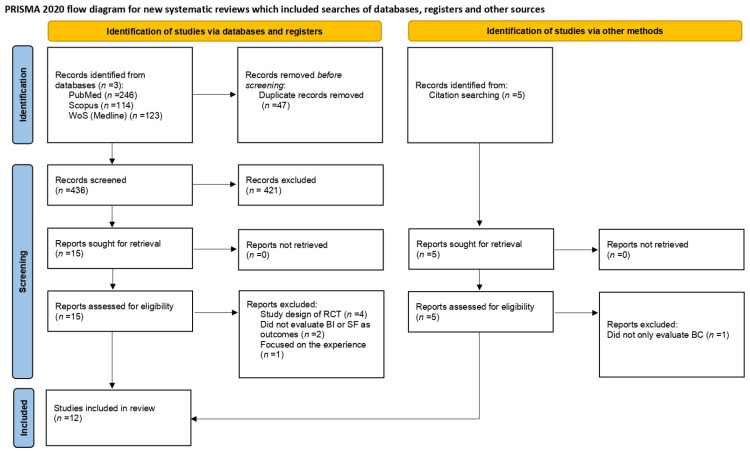
PRISMA flow diagram of the study selection process. WoS = Web of Science; BI = body image; SF = sexual functioning; BC = breast cancer.

For the present scoping review, we included Randomized Control Trials (RCT) whose interventions consisted of different types of PE and the outcome variables were body image and/or sexual health in a population affected by or who have survived BC. As the current review is the first on this topic, to gather the widest body of evidence, an a priori temporal frame of publication was not selected as an inclusion criterium. Studies using both qualitative and quantitative approaches were included. In contrast, manuscripts with different study designs, or focusing on different cancer types and those which were not written in English were excluded from the analysis. The flow diagram of the study selection process is depicted in Figure 1. Titles and abstracts of the studies were manually screened by two independent authors, who selected the studies for further screening based on the above-mentioned inclusion criteria. Afterwards, duplicates were removed. Full texts were screened by one independent author and in case any doubt arose, a comparison with two other authors was requested. From a full-text analysis, the following information was extracted: the type of intervention, sample size and age of the intervention groups, duration of the interventions, inclusion and exclusion criteria of the RCT, the methodology used to assess BI and SF, surgical treatments and therapies for BC.

## 3. Results

The research queries on the three databases yielded a total of 483 results: PubMed (*n* = 246), Scopus (*n* = 114) and Web of Science (*n* = 123). Other papers (*n* = 5) included in the review were retrieved via a manual search of the reference list of similar reviews, for a total of 488 papers. After duplicate removal, 441 papers were screened based on title/abstract. Twenty studies were assessed for eligibility and the full texts were examined. Of the twenty studies, eight were excluded; four were study designs of RCTs [27,28,29,30]; two did not rate BI or SF as outcome variables [31,32]; one focused on the experiences of exercise in BC patients [33] and one did not exclusively evaluate BC patients [34].

The remaining twelve RCTs included in this review investigated the effect of different types of PE on BI and SF. The effects of resistance training have been evaluated in two RCTs [35,36]; whereas the effects of different forms of aerobic training have been assessed in five studies [37,38,39,40,41]. Other forms of PE such as Pilates, dance, yoga and mobility exercise were utilized in four RCTs [42,43,44,45], while in the study carried out by Anderson et al. [46], which adopted a multimodal design, the PE proposed to the intervention group was not specified.

In the greater part of the RCTs included in this review, BI and SF were both evaluated [36,37,38,39,41,42,43,44]. One study exclusively evaluated BI [45], and three only evaluated SF [35,40,46].

### 3.1. The Effects of Physical Exercise on Body Image

The study proposed by Duijts et al. [37] aimed to compare the effectiveness of a cognitive behavioural therapy (CBT), PE intervention and a combination of these two methodologies on BI in a large sample of BC patients (*n* = 422; mean age: 48 years old). The PE intervention was a twelve-week, individually tailored, self-administered training lasting for approximately three hours a week. The aerobic intervention (e.g., running, cycling, walking) was agreed upon with an expert. The intensity of the aerobic training ranged between sixty and eighty percent of their reserve heart rate. The CBT intervention was structured in six group sessions of 90 min performed weekly in which relaxation exercises were included. Women which were allocated into the combined intervention group underwent both interventions simultaneously. A dropout rate of approximately 24% was reported by the authors for the PE group (*n* = 104). Forty-three patients in the PE group underwent to mastectomy and approximately 19 were in hormone therapy. In the CBT group (*n* = 109), around 88% of the group were on hormone therapy and approximatively 53% underwent to mastectomy. In the combined intervention group (*n* = 106), the percentages for hormonal therapy and surgical intervention were similar: around 51% of the group underwent to mastectomy and 90% were taking hormone therapy. Of the 103 patients composing the control group, 54 underwent to mastectomy and 86 were under hormone therapy. The hormone therapy type was not reported. The European Organisation for Research and Treatment of Cancer Quality of Life Breast Cancer questionnaire (EORTC QLQ-BR23) functional subscale was used in this study to investigate changes in BI following the different interventions. Results showed no significant intervention by time interaction effect on BI. In the study of Montagnese et al. [38], aiming to investigate the effects of a lifestyle modification on QoL in BC survivors, participants (*n* = 227; age: 50 years old) were randomly assigned to an intervention group or to a control group. Both groups adhered to the Mediterranean dietary regimen and were supplemented with vitamin D. The intervention group underwent a PE programme, comprehensively detailed in the study design published by Augustin et al. [47], which included 30 min of moderate brisk walking daily, for a year. One hundred and twenty-four survivors were on hormonal therapy. No data are avaiable about surgery type. The EORTC QLQ-BR23 Quality of Life Questionnaire Breast Cancer subscale was employed to assess possible changes in BI. After twelve months of PE intervention, BI improved significatively for all participants. Moreover, when stratified by hormone therapy, participants in hormone therapy showed lower improvement in BI than their counterparts not undergoing hormone therapy. The aim of the RCT proposed by Klavina and colleagues [39] was to investigate the effect of a high-intensity interval training (HIIT) on QoL and the side effects of chemotherapy treatments during their administration. In this study, 56 patients (mean age: 49 years old) were randomly assigned to a control group (*n* = 27), which followed the usual care provided by physicians, or to an intervention group (*n* = 29) that underwent PE. The PE intervention lasted six months and was administered two or three times per week for a total of sixty-four sessions. The PE intervention consisted of high-intensity walking, aiming to reach 85–95% of the maximum heart rate. Each session lasted thirty-four minutes and started with a warm-up of six minutes, where the intensity was progressively increased to reach 60–70% of the maximum heart rate. After this, four minutes of walking at a high intensity (85–95%) and three minutes of active rest (55–70%) were alternated four times. During exercise, heart rate was self-monitored by patients. The European Organisation for Research and Treatment of Cancer Quality of Life Breast Cancer questionnaire (EORTC QLQ-BR23) has been used in this study to assess BI. After six months of intervention, a significant worsening in BI resulted both for the intervention and control groups. The authors did not give information regarding hormone therapy and surgical treatments. The study carried out by Saarto et al. [41] consisted of a large, open, prospective RCT which proposed an aerobic intervention to BC survivors. The study lasted for twelve months, and the intervention provided a combined approach of supervised and home-based training aiming to reach at least three training session per week. Supervised training was carried out in groups once a week for one hour and step-aerobic and circuit training classes were alternated weekly. Exercise intensity ranged between fourteen and sixteen on the Borg’s rating of perceived exertion (RPE) scale. Home training could be chosen freely but had to be similar to the supervised training: activities such as Nordic walking and brisk walking were suggested. At the final analysis, a sample of 500 BC patients (mean age: 52 years old) participated in the study: 263 were allocated to the exercise intervention (I) and 237 belonged to the control group (CC). Approximately 50% of the participants assigned to both groups underwent a mastectomy (CC = 129; I = 127) as a surgical treatment, while the remaining participants (CC = 108; I = 136) underwent a resection. Approximately 85% of the participants assigned to the exercise group were on hormone therapy: half of them were receiving Tamoxifen, while the remaining patients were receiving Aromatase inhibitors. A similar rate of patients was undergoing hormone therapy in the control group. The European Organisation for Research and Treatment of Cancer (EORTC) questionnaire subscale (EORTC-QLQ-BR23) was employed to evaluate changes in BI. Two assessments were carried out (pre- and post-intervention). No significant effect of the intervention was observed for BI.

Speck et al. [36] investigated the effect of resistance training on BI in BC patients. Two hundred and thirty-four BC survivors who were both affected by lymphedema or not participated in this study. Patients were randomized into two groups: 113 patients were assigned to the intervention group (mean age: 56 years old) and 121 were allocated to the control group (mean age: 58 years old). Participants with lymphedema were equally distributed across groups. The intervention had a duration of one year with a bi-weekly frequency. Each session lasted ninety minutes. The resistance training exercises, involving the upper and lower body in every session, included three sets of ten repetitions per exercise. The authors did not report any information about surgical and hormone treatments. The Body Image and Relationships Scale (BIRS) was utilized to assess BI. The BIRS was used to assess patients at baseline and after twelve months. The authors reported a significant improvement in BI in the intervention group only [36].

In the study conducted by Sandel et al. [45], focusing on the effect of a dance movement programme on BI, 38 BC patients (mean age 61 years old, range: 38–82) were recruited: 19 of them were allocated to the control group (CC) and 19 were allocated to the intervention group (I). The intervention group underwent a dance training programme for twelve weeks. The training sessions, each lasting one hour and held bi-weekly, were divided into a warm up, core exercise, dance movement and cool-down. The Body Image Scale (BIS) was used to assess BI. Two assessments were carried out: one before the intervention and one after thirteen weeks. After thirteen weeks, BI showed a comparable improvement in both groups, with no significant time and intervention interaction. Patients included in the study conducted by Sandel et al. [45] showed a great variety of surgery types (see Table 2): half of the patients included in the intervention group underwent a total mastectomy (10 out of 19) while approximately 70% (11 out of 16) of the patients allocated to the control group underwent a partial mastectomy. In both groups, almost all patients underwent lymph node removal (CC = 14; I = 16). No information about hormone therapy was provided in this study. Boing et al. [42] compared the effects of two different approaches on BI in BC survivors. A total of 74 survivors (mean age: 55 years old) participated in the study. Twenty-five were allocated to the Pilates group, 25 were placed into the Belly dance group and twenty-four were in the control group receiving education sessions for sixteen weeks. Fifty-two participants completed the intervention (Pilates = 18; Belly dance = 18; Control = 16).

Both PE interventions were performed three times a week and every session lasted one hour. Training sessions were structured in different parts (warm-up/stretching; main part of class; cool down). The Pilates intervention started with a warm-up focusing on a breathing technique and an exercise involving the scapulo-humeral cingulum, head, arms and spine. The main part of the Pilates lesson was performed in the supine position, to avoid floor’s impact on joint, and the goal of this stage was improving limb and core mobility and strength. Further, a Thera Band and toning ball were added to progressively increase intensity. The cool down included active and passive stretching of the cervical and lower spine performed on toning balls. The belly dance intervention was structured in three parts: the warm-up included mobilization exercises starting from the upper body and progressively reaching the lower body. Movements were performed at a rhythm of 80 beat per minutes (bpm) for ten minutes. The second part was performed at a rhythm ranging between 120 and 150 bpm for about forty minutes. The last part aimed at cooling down participants and was performed at a rhythm of 80 bpm for ten minutes. Of the total sample, 16 patients underwent a total mastectomy with breast reconstruction, 12 underwent a total mastectomy without breast reconstruction and 46 had a breast-conserving surgery. During the period of the intervention, 43 patients were taking aromatase inhibitors and 31 were taking Tamoxifen. The Body Image After Breast Cancer (BIBCQ) questionnaire was employed to test the effects of the two interventions on BI. Results showed that the belly dance group displayed a significant improvement in BI (subscale of the BIBCQ) from baseline to post-intervention assessment. The RCT of Ochalek et al. [43] investigated the effect of light arm compression on QoL and BI in BC patients. Forty-five women were randomly allocated to an exercise without a sleeve, arm compression group (*n =* 22) or to an exercise with a sleeve, arm compression group (*n =* 23). The mean age was reported separately for both groups; the intervention group was significantly younger than the control group (53 ± 9.3 vs. 64 ± 8.6 years). For both groups, the proposed PE intervention included an exercise of active mobility for the upper limbs (i.e., shoulder and elbow flexion-extension; abduction, adduction and rotation of the shoulder and hand grip strength exercises) combined with diaphragmatic breathing. Patients were instructed to perform the training daily for fifteen minutes for twelve months. In this study, QoL in breast cancer was measured by the European Organization for Research and Treatment of Cancer (EORTC) QLQ-C30 questionnaire, in particular the subscale QLQ-BR23 BRSEF of functional aspects was used to assess BI. Results showed that after twelve months, BI did not differ significantly between groups. The surgical approach was heterogeneous: some patients underwent a conservative approach, while the remaining ones underwent a mastectomy.

Although it was heterogeneous, the surgical approach was equally represented in the two groups. Hormone therapy information is not available in this RCT. The study conducted by Rahmani and Talepasand [44] evaluated the effect of mindfulness and yoga training on BI in BC patients. A total of 24 BC patients (mean age: 45 years old) were randomly allocated to a control and to an intervention group. The yoga intervention was administered for two hours once a week for two months. The training sessions consisted of typical yoga combined with breathing exercises. The control group did not receive any intervention, until the end of the programme. The Specialized Supplemental Questionnaire to Measure Special “Life Quality” of Patients with Breast Cancer (QLQ-BR23) was adopted to evaluate possible changes in BI using the questionnaire’s functional subscale. Questionnaires were self-administered, and participants were asked to fill them out at three time points: pre-intervention; post-intervention and after a follow-up of two months. The results showed a significant improvement in BI in the yoga group after the intervention. Conversely, a lower BI score was registered for the control group at the same stage. This RCT did not provide any information about surgical interventions and hormone therapies.

### 3.2. The Effects of Physical Exercise on Sexual Function

In the study conducted by Pinto et al. [40], the effect of an aerobic intervention at moderate intensity on SF in BC patients was analyzed. Eighty-six patients (mean age: 54 years old) were randomly assigned to an intervention or to a control group. In the intervention group, a variety of surgery types were utilized: 27.9% underwent a lumpectomy and 48.8% underwent a lumpectomy with node dissection; 8% underwent a mastectomy, either a simple one or one with breast reconstruction. In the control group, the most used surgical treatment was a lumpectomy with node dissection (51.2%) followed by a simple mastectomy (32.6%); conversely, a simple lumpectomy (16.3%) and a mastectomy with breast reconstruction (7%) were the least used. Regarding hormone therapy, 50% of the intervention group was receiving hormone therapy, whereas in the control group, approximately 100% of patients were receiving hormone therapy. Patients assigned to the intervention group were instructed to start aerobic training twice a week for at least ten minutes for twelve weeks. In this interval, the training volume was progressively increased, aiming to reach thirty minutes daily. The activities, freely chosen by participants, had an intensity ranging between 55% and 65% of their maximum heart rate. Patients assigned to the control group were asked to maintain their habitual PA levels. The Body Esteem Scale (BES) was employed to evaluate SF. At the end of the training period, no difference emerged between the two groups. The effects of aerobic training on SF were also evaluated by four different authors, whose studies have been detailed in the previous paragraphs [37,38,39,41]. Duijts et al. [37], comparing the effect of a cognitive behavioural therapy, PE intervention and a combination of these two methodologies on SF in BC patients, showed that SF, as assessed by means of the Sexual Activity Questionnaire (SAQ), did not demonstrate any significant increase after twelve weeks of intervention in both groups. Montagnese et al. [38], evaluating the effect of a healthy lifestyle in BC patients on SF, as assessed by means of the EORTC QLQ-BR23 Quality of Life Questionnaire Breast Cancer subscale, reported a worsening in SF after twelve months of intervention, especially for BC survivors which were not on hormone therapy. Klavina and colleagues [39] administered an intervention of high-intensity walking in their study, as previously described in the BI paragraph. Results on SF, assessed using the specific subscale of the European Organisation for Research and Treatment of Cancer Quality of Life Breast Cancer questionnaire (EORTC QLQ-BR23), showed significant improvements in the intervention group after six months of PE. The same assessment tool (EORTC QLQ-BR23 Quality of Life Questionnaire Breast Cancer subscale for SF) was also utilized by Saarto et al. [41] to evaluate the effect of aerobic training on SF in BC patients. Their results showed no significant improvement after the intervention both for the intervention and control groups.

Two studies focused on the effects of resistance training on SF in BC survivors [35,36]. Ohira et al. [35] proposed weight training for BC survivors to investigate its effects on SF in BC survivors. Eighty-six patients (mean age: 54 years old) were assigned either to an intervention or to a control group. Both groups were on hormone therapy, although they were using different medications. No information was reported about surgical interventions. The intervention group received resistance training in small groups twice a week for twenty-six weeks. Exercises were performed using isotonic machines or free weights focusing on the major muscle groups and patients were instructed to perform stretching exercises before and after each strength training. After the first thirteen weeks of supervised training, a further thirteen weeks of unsupervised strength training were granted, during which participants were asked to refrain from participating in other forms of training (i.e., aerobics or multicomponent trainings). Changes in SF were evaluated by means of one of the subscales of the cancer rehabilitation evaluation system short form (CARES-SF) submitted at baseline and at the end of the intervention. Results did not show improvement in the sexual global score for the intervention group. The effect of strength training on SF in BC patients was also investigated by Speck and colleagues [36] using the Body Image and Relationships Scale (BIRS) subscale “appearance and sexuality”. The characteristics of the sample and of the training programmes have already been detailed in this review in the BI paragraph of the results section and for brevity, the information will not be replicated here. This study showed that twelve months of strength training resulted in a significant improvement in the SF subscale of the BIRS. Conversely, no significant change in SF was observed in the control group.

The studies of Boing et al. [42], Ochalek et al. [43] and Rahmani and Talepasand [44], already described in the BI paragraph of this review, evaluated effects of other types of PE interventions on SF in BC patients. The Female Sexual Function Index (FSFI) was used by Boing et al. [42] to asses SF after participation in belly dance classes. Their results showed a time-by-group interaction effect for the pain/discomfort variable, with the belly dance group showing a significant reduction from pre- to post-intervention assessments. The Quality of Life Questionnaire Breast Cancer subscale (EORTC QLQ-BR23) was employed by Ochalek et al. [43] to assess the effect of mobility exercise for the upper limbs using light arm compression on SF in BC patients. Their results showed a significant improvement in SF in the group in which PE was practiced without arm compression. Rahmani and Talepasand [44] used the Specialized Supplemental Questionnaire to Measure Special “Life Quality” (QLQ-BR23) to asses the effect of yoga training on SF in patients with BC. This study showed no significant changes in SF after the exposure to yoga training. SF is one of the outcomes of the multimodal RCT proposed by Anderson et al. [46], aimed at inducing a behavioural change acting on different aspects of women’s lives. The programme lasted twelve weeks and gave participants weekly guidelines on their daily habits to adopt a healthy lifestyle. Although the PE intervention was not clearly declared in their manuscript, the authors refer to the World Cancer Research Fund/American Institute for Cancer Research 2010 guidelines. Patients assigned to the PE intervention group were provided with a programme journal in which a weekly exercise planner was included to plan and report their activities. For this study, 51 BC patients (49 years old), randomly allocated either to an intervention group (I; *n* = 26) or to a control group (CC; *n* = 25) completed the study. Participants that were eligible for the study before the allocation underwent two different surgical approaches: a mastectomy was used for 15 women in the intervention group and for 14 in the control group, and a more conservative surgical approach (lumpectomy) was applied instead for 14 women in the intervention group and for 19 in the control group. In both groups, approximatively 85% of patients were on hormone therapy. The main instrument used in this study was the Greene Climacteric Scale (GCS), a 21-item questionnaire used to assess climacteric symptoms associated with menopausal state. The GCS questionnaire is divided in four subscales, and one of these subscales aims to evaluate SF. Patients were assessed on the GCS at baseline and at the end of the intervention. No significant effect of the proposed intervention on SF was reported.

## 4. Discussion

In this scoping review, we aimed to analyze the extant literature focusing on the effects of physical exercise (PE) interventions on body image (BI) and sexual function (SF) in woman living with and who have survived breast cancer. Twelve studies were included in this scoping review. Aerobic training was the most represented modality of PE [37,38,39,40,41], followed by resistance training [35,36], and other forms of training [42,43,44,45]. In this latter category, we included studies focusing on holistic activities (yoga, Pilates, belly dancing), dance and mobility programmes, and one study which did not describe the PE intervention used [46].

The analysis of the existing evidence showed that PE had marginal effects on BI and SF. It is worth noting that studies included in this review showed a great heterogeneity in terms of physical intervention characteristics, assessment tools used to investigate the primary outcome measure, sample characteristics and sample size. All these issues may have limited the effectiveness of the interventions based on the PE.

Physical exercise, in six out of twelve studies [35,36,41,42,44,45], was carried out in a group, which, according to White and colleagues [48], may foster adherence to PE interventions and maximize the benefits for the participants, ultimately leading to an improvement in QoL [49]. On the other hand, group exercise may imply lower supervision in terms of execution accuracy, training intensity and, broadly speaking, participant monitoring, possibly leading to the reduced efficacy of the training process compared to individual training.

Our results suggest that interventions with a short-term duration may not be able to achieve significant improvement in BI or SF, irrespective of whether the interventions focused on aerobic, resistance or other types of activities. Indeed, only one study with a short duration showed positive effects of PE on BI [44]. Regarding the studies that adopted a long-term intervention lasting more than four months, five of them reported significant improvements in BI and SF after exercise [36,38,39,42,43].

Our results showed that most of the studies focusing on aerobic exercise followed the ACSM guidelines for the prescription of PE for BC patients in terms of weekly training volume and intensity. In particular, at least 180 min of aerobic exercise were proposed, except for in the study conducted by Klavina et al. [39], in which 104 min of weekly activity were administered and in the study conducted by Saarto et al. [41], which did not specify the overall weekly training volume. Coherently, the training intensity for aerobic exercise was also in line with the ACSM guidelines, ranging from sixty to ninety-five percent of their maximum heart rate [37,39,40] or from fourteen to sixteen [41] on the rate of perceived exertion scale [50]. Conversely, following the guidelines of the American Cancer Society and the World Cancer Research Fund, Montagnese et al. [38] adopted a moderate-intensity protocol, as previously stated in the published study design [47]. The activities proposed as aerobic exercise were mainly running, cycling, brisk walking, step-aerobics, and circuit training. Notwithstanding the coherence of these studies with the ACSM, or the American Cancer Society and the World Cancer Research Fund guidelines in terms of volume and intensity, limited benefits of aerobic exercise were observed for the greater part of the studies included in this scoping review. Specifically, only one study (out of five) reported beneficial effects of aerobic activity on BI [38] and, likewise, a significant effect of aerobic exercise interventions was observed on SF in only one study [39]. As previously reported, the limited benefits of aerobic exercise may be accounted for by the heterogeneity of the training programme durations reported in the studies included in this scoping review, ranging from 12 weeks [37,40] to 12 months [38,41]. However, other factors may also account for these controversial results. Among these, the intervention modality, i.e., in sport facilities or home-based and group vs. individual training, may play a pivotal role. Four of the studies adopting an aerobic intervention were home-based and unsupervised [37,38,39,40], whereas one study [41] adopted a mixed design consisting of one supervised and two unsupervised training sessions weekly. Only in the study conducted by Montagnese et al. [38] did the aerobic intervention result in an improvement in BI. Interestingly, in this study, patients were asked to exercise at least 30 min daily, highlighting the importance of a combination of training volume and duration for successful interventions. Following the study conducted by Klavina et al. [39], the significant improvement observed in SF may be accounted for by the use of multimodal interventions. According to the authors, interventions such as sex education and counselling administered concurrently with exercise may improve SF. Unfortunately, the authors did not actually investigate this association in their sample, leaving this question unanswered. As suggested by the study conducted by Pinto et al. [40], women with BC demonstrated psychological (e.g., mood state) and physical improvements (e.g., improvements in cardiorespiratory fitness) that may confirm the observed effects on BI and SF. However, these aspects were not the same in all the above-mentioned studies. Therefore, the modality of how aerobic training is performed could have a different impact from a psychological point of view, thereby affecting physiological aspects. Contrasting findings emerged from the analysis of the interventions based on resistance training. Indeed, the two studies retrieved for this scoping review showed opposite results. Speck et al. [36] reported significant effects of resistance training on BI and SF, whereas Ohira and colleagues [35] did not observe significant effects on SF. Both groups of authors applied the same protocol and did not specify the training intensity. It is noteworthy that, concerning resistance training when proposing upper limb resistance exercises to BC survivors, critical issues may arise such as pain and discomfort as a consequence of surgical treatment. In the study conducted by Ohira et al. [35], 87% of the participants included in the intervention group underwent an axillary dissection. A complication caused by this surgery could be axillary web syndrome which, as a consequence of the formation and development of fibrotic tissue, could cause pain and functional limitation in women consequential to the surgical treatment [51], potentially limiting the effectiveness of the training programmes. Furthermore, these contrasting results could be explained both from the perspective of the difference in sample size and the intervention duration differences. Indeed, in the study conducted by Speck et al. [36], the duration of the resistance training lasted twelve months and involved a large sample size, whereas in the study conducted by Ohira et al. [35], the training protocol was shorter and involved a smaller group. These aspects advocate for a thorough description of the training protocols adopted by including fundamental information on training volume and intensity that may play a fundamental role in modifying BI. Notably, the effects of resistance training were assessed only in BC survivors, leaving a gap of knowledge on the effect of this training modality on BI and SF in BC patients. Interestingly, it could be hypothesized that resistance training may help women with BC feel more empowered and regain a sense of control over their bodies [35,36]. As they build strength and increase lean muscle mass, they may experience improvements in their overall QoL. This sense of physical power could boost their confidence, helping them feel more capable in other areas of their lives [35]. Therefore, physiological improvements could significantly affect the psychological aspect, making women with BC feel stronger both physically and mentally. The studies that adopted various forms of PE reported, in general, beneficial effects on BI and SF. Although a couple of studies failed to show a significant effect of PE [45,46], the remaining studies with different types of interventions showed significant improvements in BI [44], SF [43] or in SF and BI [42]. In this latter study, the authors reported significant improvements in BI and SF in woman with BC participating in belly dance classes. These results may be accounted for by the rhythm of the tracks used for the belly dance classes. Indeed, the beats per minute (BPM) of the tracks ranged from 80 to 150. It is likely that these high-BPM values may be associated with a high cardiovascular impact, which may ultimately lead to an improvement in body composition and to an improved perception of BI. Concerning SF, Boing et al. [42] proposed that the significant improvement observed after the belly dance intervention could be associated with the specificity of the movements performed during this activity that may positively influence the perception of comfort in some positions during sexual activities. Moreover, belly dancing, due to its characteristics may induce an improvement of the neuromuscular control of pelvic muscles leading to an improvement on SF. Additionally, this discipline, due to its artistic nature, may improve the perceived sensuality of patients, thereby enhancing their BI.

The significant improvement reported for BI after the intervention of mindfulness and yoga administered by Rahmani and Talepasand [44] could be explained considering the multidimensional construct of BI [10]. With this in mind, a multimodal intervention encompassing mindfulness and yoga exercises could reasonably be effective on the different domains constituting BI. Further, other factors may also have boosted the effectiveness of the intervention proposed in this study. In addition, a training among people experiencing the same medical condition may enable participants to feel more comfortable and accepted [33], enhancing their capacity for self-acceptance.

One of the major consequences of cancer therapies is cancer-related fatigue, which causes a strong sense of distress in women with BC. We hypothesize that this distress is strongly linked to the fear of experiencing adverse events during PE and a reduced sense of self-efficacy. These factors could lead women with BC to avoid PE, potentially compromising their health status. Consistent with previous studies [52,53], SF is a highly complex dimension influenced by various psychological (e.g., embarrassment, anxiety, shame) and physical factors (e.g., difficulty with vaginal or clitoral stimulation, poor lubrication). Menopause further complicates SF, often accompanied by a decline in PA levels, which highlights the negative impacts of menopausal symptoms [54]. In women with BC, these symptoms are often amplified, particularly due to hormonal therapies [46]. It is worth noting that women not receiving hormone therapy are more likely to show significant improvements in BI. On the other hand, patients on hormone therapy are more prone to increase their SF after PE. Poor SF may cause women with BC to perceive themselves as less attractive to partners and to increase their sense of frustration with their bodies that are already scarred by cancer-related treatments, thus further harming their body image. Therefore, studies aiming to investigate the effect of PE on patients with BC should carefully consider adding this information in their analyses.

Among the studies included in this scoping review, only five [36,39,43,44,45] did not provide information about the hormone therapies being received within their sample. In the remaining ones, participants were receiving hormone therapies of different percentages and types (i.e., tamoxifen, aromatase inhibitors, among others). It is well known that hormonal therapy is a fundamental step after different BC treatments, but this could lead to some adverse effects [55] such as body weight gains [33]. This may represent a further difficulty in women with BC assuming hormone therapy which may limit the effectiveness of PE on BI. According to the results of this review, the administration of PE has little or no effect on BI and SF in women living with and who have survived breast cancer. However, some issues arise from this scoping review. First, a thorough explanation of the characteristics of the training protocols in terms of volume, intensity, and duration is needed for a comprehensive evaluation of the effectiveness of the intervention aiming at improving BI and SF. Second, the studies focusing on aerobic programmes were mostly unsupervised. Supervised activity may allow for an enhanced monitoring of the training and of the adherence to the training programmes, compared to unsupervised ones, thus allowing for a more accurate evaluation of the effectiveness of such training programmes. This aspect shall be carefully considered as a potential source of bias when evaluating the effectiveness of these interventions.

Our study is affected by some limitations. The first limitation regards the heterogeneity of the samples analyzed in this scoping review in terms of sample size, age, the stage of BC and the characteristics of women who have survived BC that were considered. Therefore, considering the fact that we did not check for study quality, we believe that the heterogeneity of the methodology may limit the generalizabily of the results of this scoping review. For instance, the absence of standardized variables like the PE protocol duration, intensity, volume, administration and typology, despite the effects of the hormone therapy, may all increase the heterogenity of the results and limit the informative content of this study.

Furthermore, the limited number of RCTs included in our scoping review may not be totally representative of the effects of PE interventions on BI and SF. Future literature reviews encompassing larger numbers of studies shall be conducted to safely interpret the effect of PE on BI and SF.

## 5. Conclusions

In conclusion, the results of this scoping review showed that PE has marginal effects on BI and SF in women with BC and women who have survived BC. The analysis of existing evidence enabled us to identity issues related to the administration of PE in this population that need particular attention. In particular, interventions aiming at improving BI and SF should be carried out for at least six months. Based on our results, although the effects of PE are generally limited, holistic activities and dance seem to be the activities that are associated with the largest improvements. Lastly, an accurate evaluation of possible mediators of the effects of PE on BI and sexual function (i.e., hormon therapy) shall be considered before safe conclusions on the effect of PE can be drawn. Future studies should evaluate the mediating role of hormonal therapies on the effect of PE on BI.

## Figures and Tables

**Table 1:** Research queries used for three databases; last accessed 24 January 2025.

Database	Query	Results
PubMed	((((((((((Physical exercise [Title/Abstract]) OR (physical activity [Title/Abstract])) OR (training [Title/Abstract])) OR (aerobic exercise [Title/Abstract])) OR (resistance exercise [Title/Abstract])) OR (combined exercise [Title/Abstract])) AND (body image [Title/Abstract])) OR (body perception [Title/Abstract])) AND (sexual health [Title/Abstract])) OR (sexual dysfunction [Title/Abstract])) AND (breast neoplasm [MeSH Major Topic])	246
Scopus	(TITLE-ABS-KEY (“physical exercise”) OR TITLE-ABS-KEY (“physical activity”) OR TITLE-ABS-KEY (training) OR TITLE-ABS-KEY (“aerobic exercise”) OR TITLE-ABS-KEY (“resistance exercise”) OR TITLE-ABS-KEY (“combined exercise”) AND ALL (“body image”) OR ALL (“body perception”) AND ALL (“sexual health”) OR ALL (sexuality) OR ALL (“sexual dysfunction”) AND ALL (“breast neoplasm”))	114
Web of Science (Medline)	(((((TS = (physical exercise)) OR TS = (physical activity)) OR TS = (training)) OR TS = (aerobic exercise)) OR TS = (resistance exercise)) OR TS = (combined exercise) AND ((TS = (sexual health)) OR TS = (sexuality)) OR TS = (sexual dysfunction) AND (TS = (body image)) OR TS = (body perception) AND TS = (breast neoplasm)	123
Total		483

**Table 2:** Results of the included studies.

Author	Sample Size (n)	Mean Age (Years) ± (SD)	Type of Intervention	Duration Intervention	Methodology	Surgical Treatement	Hormonal Therapies	State of Disease	Main Finding
**Aerobic Training**									
Duijts et al., (2012) [37]	Tot = 422; CBT = 109; PE = 104; CBT/PE = 106; CC = 103.	Tot = 48.2 ± 5.6; CBT = 48.2 ± 5.7; PE = 47.7 ± 5.6; CBT/PE = 49.0 ± 4.9; CC = 47.8 ± 6.0	Multimodal intervention; PE: aerobic. (eg, swimming, running, cycling) 60–80% HRmax	12 weeks; 150/180 min × week.	European Organisation for Research and Treatment of Cancer Quality of Life Breast Cancer questionnaire [EORTC-QLQ-BR23; BI subscale]; Sexual activity questionnaire [SAQ]	Mastectomy Tot = 211; CBT = 58; PE = 45; CBT/PE = 54; CC = 54.	Hormone therapy, ongoing: Tot = 337; CBT = 86; PE = 93; CBT/PE = 96; CC = 86.	BC	No significant improvement in BI and SF.
Klavina et al., (2024) [39]	Tot = 56; I = 29; CC = 27.	48.56 ± 7.84	High-Intensity Interval Training Walking 85–95%HRmax	6 months. 2–3 × week; 34 min × session.	European Organisation for Research and Treatment of Cancer Quality of Life Breast Cancer questionnaire [EORTC QLQ-BR23; BI and SF subscales].	N.A.	N.A.	BC	Significant improvement in SF in intervention group. Worsening of BI in both groups.
Montagnese et al., (2020) [38]	227	52.3 ± 9.3	Multimodal intervention; PE = Moderate brisk walking	12 months; 30 min × day [at least]	European Organisation for Research and Treatment of Cancer Quality of Life Breast Cancer questionnaire [EORTC QLQ-BR23; BI and SF subscales].	N.A.	Hormone therapy: Tot = 124	BC Survivor	Significant improvement in BI for participants (particularly for those not on hormone therapy). No significant improvement in SF.
Pinto et al., (2005) [40]	Tot = 86; I = 43; CC = 43.	53.42 ± 9.08	Home-based moderate-intensity PE Choosen by participants 55–65% HRmax	12 weeks; 5 × week [at least]; 30 min × day.	Body Esteem Scale [BES]	Lumpectomy [I = 12; CC = 7] Lumpectomy with node dissection [I = 21, CC = 22] Mastectomy, simple and node dissection [I = 4, CC = 14] Mastectomy with reconstruction [I = 3; CC = 3]	Hormone treatment: [I = 21; CC = 32]	BC Survivor	No significant improvement in SF.
Saarto et al. (2009) [41]	Tot = 500; I = 263; CC = 237.	I = 52.3 CC = 52.4	Aerobics/circuit training + home training. 14–16 RPE	12 months 1 × week 60 min [supervised] 1 × week [home training]	European Organisation for Research and Treatment of Cancer Quality of Life Breast Cancer questionnaire [EORTC QLQ-BR23; BI and SF subscales].	Mastectomy: I = 127; CC = 129; Resection: I = 136; CC = 108.	Aromatase inhibitor: I = 78; CC = 80; Tamoxifen: I = 146; CC = 115.	BC Survivor	No significant improvement in BI and SF.
**Resistance Training**									
Ohira et al., (2006) [35]	Tot = 86; I = 43; CC = 43.	I = 53.3 ± 8.7 CC = 52.8 ± 7.6	Weight training 10 rep × 3 set	26 weeks; 2 × week.	Cancer rehabilitation evaluation system short form [CARES-SF]	N.A.	Tamoxifen: [I = 30; CC = 27] Anastrazole: [I = 3; CC = 5] Other: [I = 0; I = 1]	BC Survivor	No significant improvement in SF.
Speck et al., (2010) [36]	Tot = 234; I = 113; CC = 121.	56.5	Strength training 10 rep × 3 set	12 months 1–13 week [supervised] 2 × week; 90 min; 13 week–1 year [unsupervised] 2 × week	Body Image and Relationships Scale [BIRS]	N.A.	N.A.	BC Survivor	Significant improvement in BI and SF in intervention group.
**Other type of training**									
Anderson et al., (2015) [46]	Tot = 51; I = 26; CC = 25.	49.2 ± 6.2	Multimodal; PE intervention not specified.	12 weeks; 150/180 min × week.	Greene Climacteric Scale [GCS]	Lumpectomy: [I = 14; CC = 19] Mastectomy: [I = 15; CC = 14]	Hormone therapy: [I = 23; CC = 22]	BC	No significant improvement in SF.
Boing et al., (2023) [42]	Tot = 52; P = 18; BD = 18; CC = 16;	55 ± 10	Pilates: - Strength exercise upper–lower limbs/core. - Mobility exercise upper–lower limbs. - Breathing techniques. Belly dance: - motor coordination. - rhythm. - Mobility exercise upper–lower limbs. Intensity progressively increased.	16 weeks; 3 × week; 60 min.	Body Image After Breast Cancer (BIBCQ); Female Sexual Function Index (FSFI).	Mastectomy with reconstruction: Tot = 16; Mastectomy without reconstruction: Tot = 12; Breast conserving surgery: Tot = 46.	Hormone Aromatase inhibitors: Tot = 43; Tamoxifen: Tot = 31.	BC Survivor	Significant improvement in BI and SF in belly dance group.
Ochalek et al., (2018) [43]	Tot = 45; I = 23; CC = 22.	I = 52.9 ± 9.3 CC = 64 ± 8.6	CC: Active upper limb mobility exercise + diaphragmatic breathing I: Active upper limb mobility exercise + diaphragmatic breathing plus compression	12 months; 15 min × day.	European Organisation for Research and Treatment of Cancer Quality of Life Breast Cancer questionnaire [EORTC QLQ-BR23; BI and SF subscales].	Breast conserving surgery: [I = 13; CC = 14] Mastectomy: [I = 10; CC = 8].	N.A.	BC	Significant improvement in SF in intervention group. No significant improvement in BI.
Rahmani & Talepasand (2015) [44]	Tot = 24; I = 12; CC = 12.	I = 43.25 ± 3.07 CC = 44.8 ± 3.28	Multimodal intervention: Mindfulness and Conscious yoga	2 months; 1 × week; 2-h.	European Organisation for Research and Treatment of Cancer Quality of Life Breast Cancer questionnaire [EORTC QLQ-BR23; BI and SF subscales].	N.A.	N.A.	BC	Significant improvement in BI in intervention group; no improvement in SF.
Sandel et al., (2005) [45]	Tot = 38; I = 19; CC = 19.	61	Movement and dance programme: - Warm-up (10/15 min) - Dance movements (25/30 min). - Cool down (10 min).	12 weeks; 2 × week [week 1–6]; 1 × week [week 6–12].	Body Image Scale [BIS]	Mastectomy [I = 10; CC = 6] Partial mastectomy [I = 8; CC = 11] Lumpectomy [I = 1; CC = 2] Lymph node removal [I = 16, CC = 14] Breast reconstruction [I = 5; CC = 3]	N.A.	BC Survivor	No significant improvements in BI.

Tot = Total; PE = Physical Exercise; N.A. = Not Assessed; CBT = cognitive behavioural therapy; I = intervention group; CC = control group; BC = breast cancer; PE = physical exercise; BI = body image; SF = sexual functioning.

## Data Availability

Data sharing is not applicable. No new data were created or analyzed in this study.

## References

[1] WHO (2024). Breast Cancer. https://www.who.int/news-room/fact-sheets/detail/breast-cancer.

[2] Bray F., Laversanne M., Sung H., Ferlay J., Siegel R.L., Soerjomataram I., Jemal A. (2024). Global cancer statistics 2022: GLOBOCAN estimates of incidence and mortality worldwide for 36 cancers in 185 countries. CA Cancer J. Clin..

[3] Veronesi U., Boyle P., Goldhirsch A., Orecchia R., Viale G. (2005). Breast cancer. Lancet.

[4] Mokhatri-Hesari P., Montazeri A. (2020). Health-related quality of life in breast cancer patients: Review of reviews from 2008 to 2018. Health Qual. Life Outcomes.

[5] van Leeuwen M., Husson O., Alberti P., Arraras J.I., Chinot O.L., Costantini A., Darlington A.S., Dirven L., Eichler M., Hammerlid E.B. (2018). Understanding the quality of life (QOL) issues in survivors of cancer: Towards the development of an EORTC QOL cancer survivorship questionnaire. Health Qual. Life Outcomes.

[6] Rosen R., Brown C., Heiman J., Leiblum S., Meston C., Shabsigh R., Ferguson D., D’Agostino R. (2000). The female sexual function index (Fsfi): A multidimensional self-report instrument for the assessment of female sexual function. J. Sex Marital Ther..

[7] Symonds T., Boolell M., Quirk F. (2005). Development of a questionnaire on sexual quality of life in women. J. Sex Marital Ther..

[8] Quinn-Nilas C., Benson L., Milhausen R.R., Buchholz A.C., Goncalves M. (2016). The Relationship Between Body Image and Domains of Sexual Functioning Among Heterosexual, Emerging Adult Women. Sex. Med..

[9] Cash T.F., Fleming E.C. (2002). The impact of body image experiences: Development of the body image quality of life inventory. Int. J. Eat. Disord..

[10] Woertman L., van den Brink F. (2012). Body image and female sexual functioning and behavior: A review. J. Sex Res..

[11] Candy B., Jones L., Vickerstaff V., Tookman A., King M. (2016). Interventions for sexual dysfunction following treatments for cancer in women. Cochrane Database Syst. Rev..

[12] Scotto L., Pizzoli S.F.M., Marzorati C., Mazzocco K., Pravettoni G. (2024). The impact of prophylactic mastectomy on sexual well-being: A systematic review. Sex. Med. Rev..

[13] Bokaie M., Firouzabadi O., Joulaee A. (2022). The effectiveness of group problem-solving therapy on women’s sexual function and satisfaction after mastectomy surgery. BMC Women’s Health.

[14] Schover L.R., Rhodes M.M., Baum G., Adams J.H., Jenkins R., Lewis P., Jackson K.E. (2011). Sisters Peer Counseling in Reproductive Issues after Treatment (SPIRIT): A peer counseling program to improve reproductive health among African American breast cancer survivors. Cancer.

[15] Hersant B., Werkoff G., Sawan D., Sidahmed-Mezi M., Bosc R., la Padula S., Kalsoum S., Ouidir N., Meningaud J.P., Belkacemi Y. (2020). Carbon dioxide laser treatment for vulvovaginal atrophy in women treated for breast cancer: Preliminary results of the feasibility EPIONE trial. Ann. Chir. Plast. Esthet..

[16] Crean-Tate K.K., Faubion S.S., Pederson H.J., Vencill J.A., Batur P. (2020). Management of genitourinary syndrome of menopause in female cancer patients: A focus on vaginal hormonal therapy. Am. J. Obstet. Gynecol..

[17] Advani P., Brewster A.M., Baum G.P., Schover L.R. (2017). A pilot randomized trial to prevent sexual dysfunction in postmenopausal breast cancer survivors starting adjuvant aromatase inhibitor therapy. J. Cancer Surviv..

[18] Juraskova I., Jarvis S., Mok K., Peate M., Meiser B., Cheah B.C., Mireskandari S., Friedlander M. (2013). The Acceptability, Feasibility, and Efficacy (Phase I/II Study) of the OVERcome (Olive Oil, Vaginal Exercise, and MoisturizeR) Intervention to Improve Dyspareunia and Alleviate Sexual Problems in Women with Breast Cancer. J. Sex. Med..

[19] Torres-Balanzá S., Fuentes-Aparicio L., Mena-del Horno S., Martínez-Aspas A., Sempere-Rubio N. (2023). Sexual Perception in Spanish Female Breast Cancer Survivors. Cross-Sectional Survey. Clin. Breast Cancer.

[20] Caspersen C.J., Powell K.E., Christenson G.M. (1985). Physical activity, exercise, and physical fitness: Definitions and distinctions for health-related research. Public Health Rep..

[21] Schmitz K.H. (2020). Exercise Oncology: Prescribing Physical Activity Before and After a Cancer Diagnosis. Exercise Oncology: Prescribing Physical Activity Before and After a Cancer Diagnosis.

[22] Campbell K.L., Neil S.E., Winters-Stone K.M. (2012). Review of exercise studies in breast cancer survivors: Attention to principles of exercise training. Br. J. Sports Med..

[23] Cramer H., Lauche R., Klose P., Lange S., Langhorst J., Dobos G.J. (2017). Yoga for improving health-related quality of life, mental health and cancer-related symptoms in women diagnosed with breast cancer. Cochrane Database Syst. Rev..

[24] Kirkham A.A., Bland K.A., Sayyari S., Campbell K.L., Davis M.K. (2016). Clinically Relevant Physical Benefits of Exercise Interventions in Breast Cancer Survivors. Curr. Oncol. Rep..

[25] Bertoli J., Bezerra E.D., Winters-Stone K.M., Alberto Gobbo L., Freitas I.F. (2023). Mat Pilates improves lower and upper body strength and flexibility in breast cancer survivors undergoing hormone therapy: A randomized controlled trial (HAPiMat study). Disabil. Rehabil..

[26] Tricco A.C., Lillie E., Zarin W., O’Brien K.K., Colquhoun H., Levac D., Moher D., Peters M.D.J., Horsley T., Weeks L. (2018). PRISMA extension for scoping reviews (PRISMA-ScR): Checklist and explanation. Ann. Intern. Med..

[27] Boing L., do Bem Fretta T., de Carvalho Souza Vieira M., Pereira G.S., Moratelli J., Sperandio F.F., Bergmann A., Baptista F., Dias M., de Azevedo Guimarães A.C. (2020). Pilates and dance to patients with breast cancer undergoing treatment: Study protocol for a randomized clinical trial—MoveMama study. Trials.

[28] Duijts S.F.A., Oldenburg H.S.A., van Beurden M., Aaronson N.K. (2009). Cognitive behavioral therapy and physical exercise for climacteric symptoms in breast cancer patients experiencing treatment-induced menopause: Design of a multicenter trial. BMC Women’s Health.

[29] Pinto B.M., Clark M.M., Maruyama N.C., Feder S.I. (2003). Psychological and fitness changes associated with exercise participation among women with breast cancer. Psycho-Oncology.

[30] Schmitz K.H., Troxel A.B., Cheville A., Grant L.L., Bryan C.J., Gross C.R., Lytle L.A., Ahmed R.L. (2009). Physical activity and lymphedema (the PAL trial): Assessing the safety of progressive strength training in breast cancer survivors. Contemp. Clin. Trials.

[31] Gokal K., Wallis D., Ahmed S., Boiangiu I., Kancherla K., Munir F. (2016). Effects of a self-managed home-based walking intervention on psychosocial health outcomes for breast cancer patients receiving chemotherapy: A randomised controlled trial. Support. Care Cancer.

[32] Joaquim A., Amarelo A., Antunes P., Garcia C., Leão I., Vilela E., Teixeira M., Duarte B., Vieira M., Afreixo V. (2024). Effects of a Physical Exercise Program on Quality of Life and Physical Fitness of Breast Cancer Survivors: The MAMA_MOVE Gaia After Treatment Trial. Psychol. Health Med..

[33] Emslie C., Whyte F., Campbell A., Mutrie N., Lee L., Ritchie D., Kearney N. (2007). “I wouldn’t have been interested in just sitting round a table talking about cancer”; exploring the experiences of women with breast cancer in a group exercise trial. Health Educ. Res..

[34] Berglund G., Bolund C., Gustafsson U.-L., Sjödén P.-O. (1994). A randomized study of a rehabilitation program for cancer patients: The ‘starting again’ group. Psycho-Oncology.

[35] Ohira T., Schmitz K.H., Ahmed R.L., Yee D. (2006). Effects of weight training on quality of life in recent breast cancer survivors: The weight training for breast cancer survivors (WTBS) study. Cancer.

[36] Speck R.M., Gross C.R., Hormes J.M., Ahmed R.L., Lytle L.A., Hwang W.T., Schmitz K.H. (2010). Changes in the body image and relationship scale following a one-year strength training trial for breast cancer survivors with or at risk for lymphedema. Breast Cancer Res. Treat..

[37] Duijts S.F.A., van Beurden M., Oldenburg H.S.A., Hunter M.S., Kieffer J.M., Stuiver M.M., Gerritsma M.A., Menke-Pluymers M.B.E., Plaisier P.W., Rijna H. (2012). Efficacy of cognitive behavioral therapy and physical exercise in alleviating treatment-induced menopausal symptoms in patients with breast cancer: Results of a randomized, controlled, multicenter trial. J. Clin. Oncol..

[38] Montagnese C., Porciello G., Vitale S., Palumbo E., Crispo A., Grimaldi M., Calabrese I., Pica R., Prete M., Falzone L. (2021). Quality of life in women diagnosed with breast cancer after a 12-month treatment of lifestyle modifications. Nutrients.

[39] Klavina A., Ceseiko R., Campa M., Jermolenko G.F., Eglitis K., Llorente A., Linē A. (2024). The Effect of High-Intensity Interval Training on Quality of Life and Incidence of Chemotherapy Side Effects in Women with Breast Cancer. Integr. Cancer Ther..

[40] Pinto B.M., Frierson G.M., Rabin C., Trunzo J.J., Marcus B.H. (2005). Home-based physical activity intervention for breast cancer patients. J. Clin. Oncol..

[41] Saarto T., Penttinen H.M., Sievänen H., Kellokumpu-Lehtinen P.L., Hakamies-Blomqvist L., Nikander R., Huovinen R., Luoto R., Kautiainen H., Järvenpää S. (2012). Effectiveness of a 12-month exercise program on physical performance and quality of life of breast cancer survivors. Anticancer Res..

[42] Boing L., de Bem Fretta T., Stein F., Lyra V.B., Moratelli J.A., da Silveira J., dos Santos Saraiva P.S., Bergmann A., Lynch B.M., de Azevedo Guimarães A.C. (2023). Can mat Pilates and belly dance be effective in improving body image, self-esteem, and sexual function in patients undergoing hormonal treatment for breast cancer? A randomized clinical trial. Arch. Women’s Ment. Health.

[43] Ochalek K., Gradalski T., Szygula Z., Partsch H. (2018). Physical Activity with and Without Arm Sleeves: Compliance and Quality of Life after Breast Cancer Surgery—A Randomized Controlled Trial. Lymphat. Res. Biol..

[44] Rahmani S., Talepasand S. (2015). The effect of group mindfulness-based stress reduction program and conscious yoga on the fatigue severity and global and specific life quality in women with breast cancer. Med. J. Islam Repub. Iran.

[45] Sandel S.L., Judge J.O., Landry N., Faria L., Ouellette R., Majczak M. (2005). Dance and Movement Program Improves Quality-of-Life Measures in Breast Cancer Survivors. Cancer Nurs..

[46] Anderson D.J., Seib C., Mccarthy A.L., Yates P., Porter-Steele J., Mcguire A., Young L. (2015). Facilitating lifestyle changes to manage menopausal symptoms in women with breast cancer: A randomized controlled pilot trial of The Pink Women’s Wellness Program. Menopause.

[47] Augustin L.S.A., Libra M., Crispo A., Grimaldi M., de Laurentiis M., Rinaldo M., D’Aiuto M., Catalano F., Banna G., Ferrau’ F. (2017). Low glycemic index diet, exercise and vitamin D to reduce breast cancer recurrence (DediCa): Design of a clinical trial. BMC Cancer.

[48] White J.L., Ransdell L.B., Vener J., Flohr J.A. (2005). Factors related to physical activity adherence in women: Review and suggestions for future research. Women Health.

[49] Pudkasam S., Feehan J., Talevski J., Vingrys K., Polman R., Chinlumprasert N., Stojanovska L., Apostolopoulos V. (2021). Motivational strategies to improve adherence to physical activity in breast cancer survivors: A systematic review and meta-analysis. Maturitas.

[50] Borg G. (1998). Borg’s Perceived Exertion and Pain Scales.

[51] Lippi L., de Sire A., Losco L., Mezian K., Folli A., Ivanova M., Zattoni L., Moalli S., Ammendolia A., Alfano C. (2022). Axillary Web Syndrome in Breast Cancer Women: What Is the Optimal Rehabilitation Strategy after Surgery? A Systematic Review. J. Clin. Med..

[52] Arias-Castillo L., García L., García-Perdomo H.A. (2023). The complexity of female orgasm and ejaculation. Arch. Gynecol. Obstet..

[53] Kontula O., Miettinen A. (2016). Determinants of female sexual orgasms. Socioaffect. Neurosci. Psychol..

[54] Cerulli C., Moretti E., Parisi A., Tranchita E., Di Lauro M., Minganti C., Perrone M.A., Murri A., Greco F., Marrone G. (2023). Correlation between physical activity, nutritional intake, and osteoporosis in postmenopausal women: A preliminary evaluation. Eur. Rev. Med. Pharmacol. Sci..

[55] Gan L., Miao Y.M., Dong X.J., Zhang Q.R., Ren Q., Zhang N. (2023). Investigation on sexual function in young breast cancer patients during endocrine therapy: A latent class analysis. Front. Med..

